# Serratus Posterior Superior Intercostal Plane Block: A Technical Report on the Description of a Novel Periparavertebral Block for Thoracic Pain

**DOI:** 10.7759/cureus.34582

**Published:** 2023-02-03

**Authors:** Serkan Tulgar, Bahadır Ciftci, Ali Ahiskalioglu, Bora Bilal, Bayram U Sakul, Ali O Korkmaz, Nureda N Bozkurt, Alessandro De Cassai, Augusto J. Torres, Hesham Elsharkawy, Haci A Alici

**Affiliations:** 1 Anesthesiology, Samsun University, Samsun, TUR; 2 Anesthesiology and Reanimation, Istanbul Medipol University, Istanbul, TUR; 3 Clinical Research, Development, and Design Application and Research Center, Ataturk University School of Medicine, Erzurum, TUR; 4 Anesthesiology and Reanimation, Ataturk University School of Medicine, Erzurum, TUR; 5 Anesthesiology and Reanimation, Kahramanmaras Sutcu Imam University, Kahramanmaras, TUR; 6 Anatomy, Istanbul Medipol University, Istanbul, TUR; 7 Anesthesiology, University of Padova, Padova, ITA; 8 Anesthesiology and Reanimation, Pain and Healing Center MetroHealth, Cleveland, USA; 9 Anesthesiology, Cleveland Clinic, Cleveland, USA; 10 Algology, Medipol University Faculty of Medicine, Istanbul, TUR

**Keywords:** serratus posterior superior intercostal plane block, thoracic analgesia, interfascial plane block, cadaveric study, pain management

## Abstract

Background and objective

We report a novel block technique aimed to provide thoracic analgesia: the serratus posterior superior intercostal plane (SPSIP) block.

Design

A cadaveric evaluation along with a retrospective case series evaluating the potential analgesic effect of the SPSIP block. This study included one unembalmed cadaver and five patients.

Interventions

Bilateral ultrasound-guided SPSIP block was used on cadavers with 30 mL of methylene blue 0.5% on each side; single-injection SPSIP blocks were used in patients. To measure results, dye spread was used in the cadaver, and dermatomal/pain score evaluation was used in patients.

Main results

Anatomical investigation in one unembalmed cadaver shows that its mechanism of action covers the rhomboid major muscle, erector spinae muscle, the deep fascia of the subscapularis/serratus anterior muscles, and intercostal nerves. In our patients, SPSIP resulted in an almost complete sensory block in the back of the neck, shoulder, and hemithorax.

Conclusion

Our cadaveric study shows extensive dye spread from C7 to T7. Patients who were administrated SPSIP block reported consistent dermatomal blockade from C3 to T10 levels of the hemitorax. The SPSIP block seems to be a safe, simple, and effective technique for thoracic analgesia.

## Introduction

Thoracic epidural and paravertebral blocks are regarded as the gold standard in thoracic surgery analgesia; ultrasonography-guided interfascial plane blocks have become increasingly popular owing to their minimal invasiveness and associated low complications [1]. Erector spinae plane block (ESPB) and rhomboid intercostal block (RIB) are interfascial blocks applied from the peri-paravertebral region and are utilized for a variety of purposes, including perioperative pain control and the analgesia management of chronic pain [1].

ESPB is a technique that has been the topic of countless publications and can be applied at multiple levels, from the cervical to the sacral vertebrae [2]. However, cadaveric and radiographic studies on the craniocaudal, lateral, and ventral dissemination of ESPB have yielded contradictory results, and clinical outcomes are unpredictable [2]. On the other hand, the extent and clinical effects of a RIB are more predictable and consistent due to better spread in the interfascial space (fewer tissue obstacles) and consistent spread to the dorsal rami [3]. However, the effect of the RIB cannot cover to the cephalic aspect of the T2 dermatome as the tissue plane deep to the rhomboid muscles does not extend above the second rib. The rhomboid muscles consist of two groups: the rhomboid major (RM) and minor (Rm). RM originates from T2-T5 spinous processes, while Rm originates from C7-T1 spinous processes and is inserted into the medial edge of the scapula.

When the muscles to the medial of the scapula are examined from cephalad to caudal, the trapezius (TM), RM, and intercostal muscles form the middle and lower muscle groups, while the TM, Rm, and serratus posterior superior muscles (SPSM) lie on the superomedial side, from superficial to deep. The SPSM originates from C7-T2 (sometimes T3) spinous processes, progressing obliquely and inserting on the lateral of the second to fifth ribs' angles. SPSM differs anatomically from the aforementioned muscles in that it is the only muscle that originates from the spinous process and extends deeply into the scapula. Due to this structure, it may theoretically be advantageous for local anesthetic diffusion to dorsal ramus and lateral cutaneous branches of intercostal nerves at C3-T7 levels.

On the basis of these anatomical features, a local anesthetic (LA) delivered to the plane between the SPSM and the intercostal muscles would diffuse to a different area from other paraspinal interfascial plane blocks. The aim of this paper was to present the results of the cadaveric evaluation of the serratus posterior superior intercostal plane (SPSIP) block, as well as our clinical applications.

## Materials and methods

Method

Cadaveric examination in one cadaver was performed following approval from Istanbul Medipol University Ethics and Research Committee (Date: 13/04/2022, No: 327). The same committee also approved (Date: 11/05/2022, No: 437) the retrospective evaluation of five patients that underwent SPSIP between 15/04/2022 and 11/05/2022. All patients gave written informed consent for inclusion of their data in this study.

Description of SPSIP block

**Figure 1:**
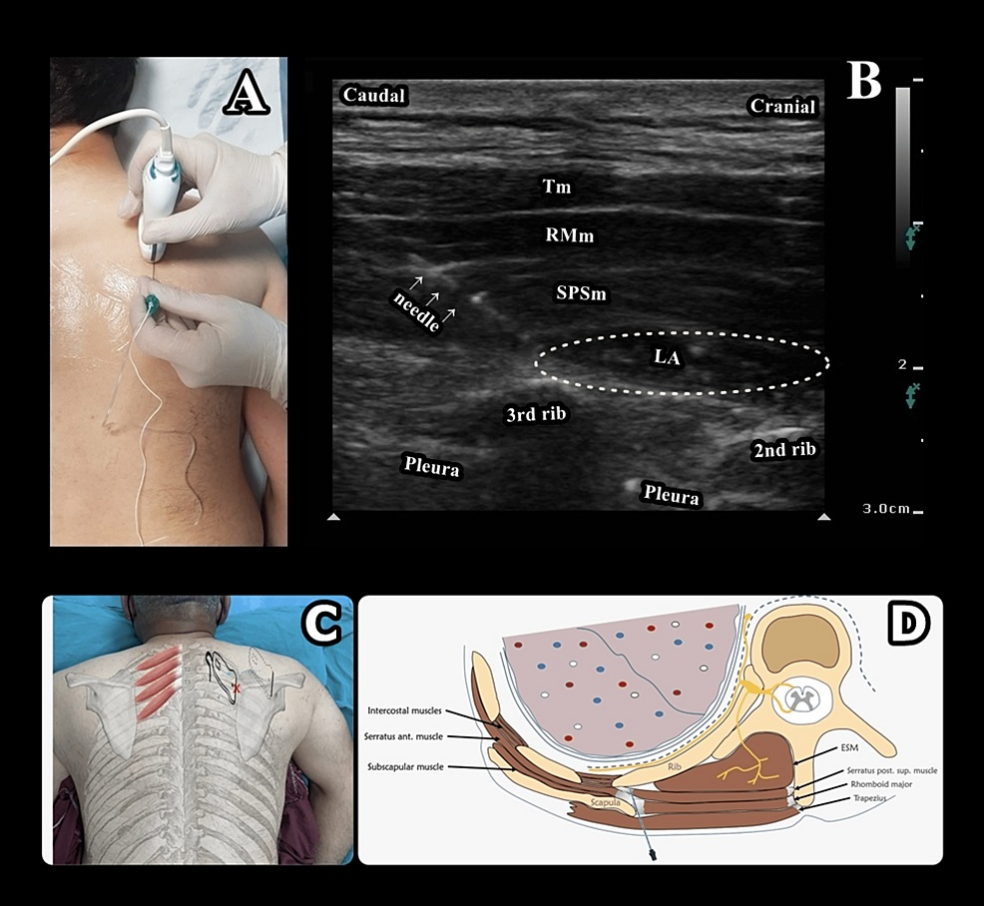
Descriptive features of SPSIP Figure 1A: Patient, probe, and needle position during SPSIP; Figure 1B: Sonoanatomy and spread of LA during SPSIP. White arrows indicate needle; Figure 1C and D: Schematic illustration at the level of the third rib demonstrating needle/probe position and injectate spread during SPSIP. SPSIP - serratus posterior superior intercostal plane, Tm - trapezius muscle, RMm - rhomboid major muscle, SPSm - serratus posterior superior muscle, LA - local anesthetic, ESM - erector spinae muscle

Although we prefer the prone position for the block, it can also be performed in the sitting position. To lateralize the scapula, the patient is instructed to grip the opposing shoulder with the hand on the side where the block is to be performed or to put the affected arm into adduction and internal rotation. A high frequency (4-12 MHz) linear transducer (B-Braun, Philips, Xperius, USA) is placed at the spinae scapula level in the transverse plane, and the upper medial border of the scapula, the trapezius muscle, Rm, SPSM and the second and third ribs are visualized. The ultrasound probe is rotated 90 degrees in a parasagittal orientation from the posterior aspect of the supraclavicular fossa to identify the first rib. After its identification, the second and third ribs are confirmed. The linear transducer is then rotated so that an oblique visualization is obtained (inferomedial-superolateral) with the upper medial border of the scapula in the sonographic view (Figure 1A). The needle (Stimuplex® Ultra 360®, B-Braun, Melsungen, Germany) is then advanced immediately medial to the scapula, aiming for the area between the second and third ribs in order to reach the fascial plane between the SPSM and intercostal muscles, either in the in-plane (caudal to cephalad) or out of the plane technique. After contact of the needle with the rib gently, 1-2mL of saline is used to confirm the correct plane, and a total of 30 mL of local anesthetic (LA) agent is administered to the superficial to the intercostal muscle. The LA should be visible, spreading cephalad and caudad over successive ribs in the interfascial plane (Figure 1B). Schematic illustration at the level of third rib demonstrating needle/probe position and injectate spread during SPSIP is seen in Figures 1C, D.

Anatomic study of the SPSIP block

Bilateral SPSIP block was performed to an unembalmed cadaver. There were no surgical incisions or scars in the paraspinal region and thorax/neck/shoulder/upper abdominal regions of the cadaver, nor were there any anatomical deformities on inspection. The cadaver was maintained at room temperature for 12 hours prior to block application. Thirty mL of methylene blue 0.5% was administered to each side using the in-plane technique (ST-HAA). One hour after administration, an experienced anatomist (AF) began dissection. Beginning at C7 and proceeding dorsally to T10, the skin, and fascia were dissected from the midline. Both sides were simultaneously dissected and compared with each other. The trapezius muscle, latissimus dorsi, rhomboids, and erector spinae muscles were dissected and evaluated for the presence of dye. After dissecting the rhomboid muscles away from the midline, the scapula and its superficial and deep muscles were shifted laterally without making a transverse incision in order to analyze the distribution of dye along the intercostal muscles. The intercostal muscles were subsequently dissected in the sagittal plane to assess the presence of dye within the muscles. To evaluate dye distribution, the deep fascia of the trapezius muscle was traced to the scapula. To define the anterior boundary of dye spread, vertical incisions were made at the posterior, middle, and anterior axillary lines.

Case series

Considering the cadaveric findings, the SPSIP block application was chosen for eligible individuals who were likely to benefit from this new block technique. All of these patients were selected from patients who were diagnosed with myofascial pain syndrome (MPS), which causes complaints in the cervical and interscapular region, according to MPS diagnostic criteria, and who had previously been treated with medications, physiotherapy, and neural therapy for this, but could not provide sufficient pain relief.

After informing patients of the risks, benefits, and alternatives of this procedure, all patients provided informed written consent. The block was performed in the prone position under sterile conditions and after administration of 2% lidocaine for local anesthetic infiltration of the skin. Dermatomal evaluation was performed 40 minutes after block administration using the cold test.

## Results

Anatomic evaluation

**Figure 2:**
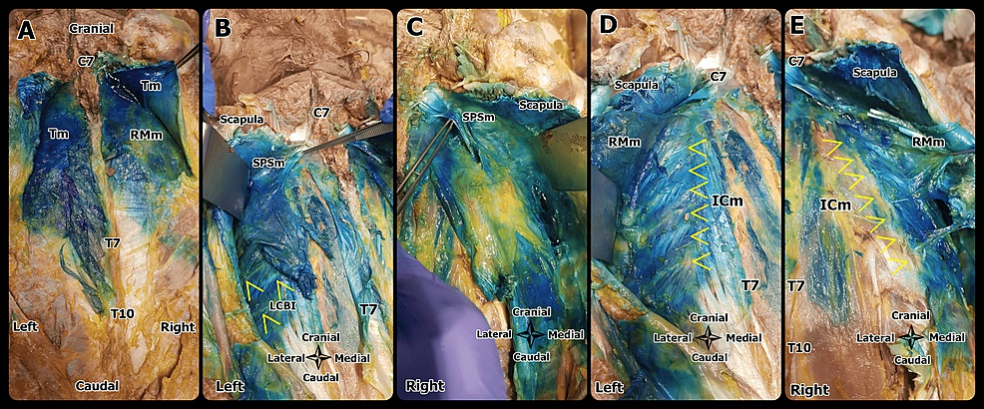
Cadaveric dissection of SPSIP Figure 2A: Dye spread is seen around Tm and RMm, Figure 2B, 2C: Dye spread is seen around SPSm under the scapula. LCBI is indicated with yellow arrows; Figure 2D, 2E: Dye spread is seen around ICm under RMm. Yellow arrows indicate ICm. Tm - trapezius muscle, RMm - rhomboid major muscle, SPSm - serratus posterior superior muscle, LCBI - lateral cutaneous branches of intercostal nerves, ICm - intercostal muscle

There was dye staining between the seventh and 10th intercostal levels along the deep fascia of the trapezius muscle on the left of the cadaver, and no staining was observed in this plane on the right side of the cadaver. Dye was present deep in the trapezius muscle, bilaterally. Dye was present in both the superficial and deep areas of the rhomboid major muscle, while it was observed in only the deep areas of the rhomboid minor muscle. Dye was observed both on the superficial and deep planes of the erector spinae muscles, bilaterally. On the intercostal muscle plane, the dye was evident bilaterally from C7 to T7, spreading to the anterior axillary line on the right and the mid-axillary line on the left. The deep fascia of the subscapularis and serratus anterior muscles were stained bilaterally. A review of dye staining observed from the cadaveric evaluation is shown in Table 1. When the deep fascia of the trapezius muscle was followed to the pars clavicularis insertion point, the dye was observed to have spread to the scapula on the left side. Spread to the brachial plexus and clavipectoral fascia could not be evaluated as neck dissection could not be performed. Cadaveric evaluation images are shown in Figure 2.

**Table 1:** Demographic information and block evaluation results for all participants NRS - pain numeric rating scale

Descriptives	Patient No	1	2	3	4	5
	Age / gender	26 / F	24 / F	45 / F	46 / F	42 / M
	Height (cm) / weight (kg)	158 / 62	160 / 65	155 / 50	158 / 55	182 / 95
	Indication	Myofascial pain syndome	Myofascial pain syndrome	Myofascial pain syndrome	Myofascial pain syndrome	Myofascial pain syndrome
Block application	Application	Left in-plane	Bilateral in-plane	Right in-plane	Bilateral in-plane	Left in-plane
	Volume (mL)	30	40	20	40	30
	Content	0.25% bupivacaine with 8mg of dexamethasone and 40mg methylprednisolone	0.25% methylprednisolone	0.25% bupivacaine with 20 mg methylprednisolone	0.25% bupivacaine with 40 mg methylprednisolone	0.25% bupivacaine with 40 mg methylprednisolone
Sensory block findings	Paraspinal	+	+	+	+	+
	Posterolateral	+	+	+	+	+
	Anterolateral	+	+	+	+	+
	Anteromedial	+	+	+	-	+
	Shoulder anterior	+	+	+	+	+
	Shoulder posterior	+	+	+	+	+
	Axilla	+	+	+	+	+
	Dermatomal coverage	C3-T7	C3–T7	C3-T10	C3-T9	C3-T9
	NRS change	8 → 0	8→0 (R) 8→3 (L)	9→0	9→0 (R) 9→1 (L)	8→1

Patients

SPSIP block was administered to a total of five patients aged between 26 and 64 years. Two of the blocks were bilateral, while the remaining four were unilateral, with a total of eight blocks being performed. Five patients diagnosed with MPS were evaluated. None of the patients had cervical spinal pathology. These patients have received non-pharmacological and pharmacological conservative treatments for MPS, but short-term pain relief was provided. Analgesia management was not achieved in the entire aching area in any of the patients.

**Figure 3:**
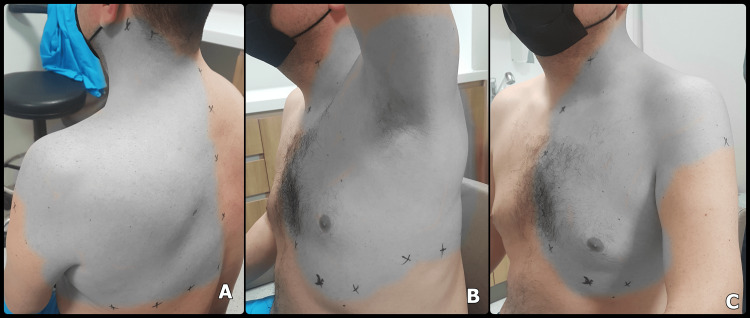
Dermatomal coverage of SPSIP A, B, and C demonstrate sensorial blockage in patient five (max coverage) SPSIP - serratus posterior superior intercostal plane

The demographic and block characteristics of the patients are detailed in Table 2. The dermatome analysis results following the SPSIP block are illustrated in detail in Figure 3. Consequently, the sensory block was found in the back, neck, shoulder, axilla, and lateral chest in all participants, and parasternal involvement was observed in 5/8 of them. Dermatomal analysis of the patients showed sensory block reached the C3 dermatome in all patients in the cephalic direction. In the cadual direction, it was determined that sensory block reached at least the T7 dermatome in all patients. We followed the patients for five weeks, and their pain numeric rating scale (NRS) scores were <4.

**Table 2:** Findings from cadaveric evaluation of SPSIP N/A - not applicable, RM - rhomboid muscle, ESM - erector spinae muscle, SPSIP - serratus posterior superior intercostal plane

Area	Right	Left
Between trapezius and RM	++	+++
Between trapezius and RM	N/A	N/A
Superficial and deep ESM plane	N/A	+
Superficial of SPSM	+++	++
Deep to SPSM	+++	+++
Between back muscles and intercostal muscles	+++ 1st to seventh intercostals	+++ first to seventh intercostal
In to intercostal muscles or more deeper	N/A	N/A
Medial and lateral border of dye in the intercostal plane	From the spinous process to the anterior axillary line	From the spinous process to the mid-axillary line
Cephalic and caudal border of dye	C7 to T7	C7 to T7

## Discussion

The cadaveric examination demonstrated that the dye injected between the serratus posterior superior muscle and the intercostal muscles spread from the deep of the subscapularis and serratus anterior muscles to the anterior axillary line and from the first rib to the seventh and eighth ribs in the craniocaudal planeIn our patients, SPSIP resulted in an almost complete sensory block in the back of the neck, shoulder, and hemithorax.

Over the past decade, interfacial plane blocks have gained prominence in regional anesthetic practices. Numerous interfascial blocks have been defined in recent years with the intention of providing effective sensory blockage in the target location by blocking the posterior-lateral or anterior branches of the spinal nerves alone or in multiples [1]. It can be determined that many new blocks, hypothesized on anatomical bases and demonstrated by cadavers/patients, are insufficient in the hypothesized target later on. For example, in cases where interpectoral block (PECs I), first defined for perioperative analgesia in patients undergoing breast surgery-is insufficient, it is combined with pesto-serratus block (PECs II) [4,5]. RIB, a more recently described block, does not provide dermatomal blockage over the third thoracic dermatome and misses the axillary and cervical area [3-6]. The distribution of the local anesthetic and the mechanism of action of ESPB remain under debate, and its clinical results vary [2,7-9]. While we predicted that the SPSIP block would cover areas that RIB misses in the cephalic direction, cadaveric data and dermatomal analyses revealed that the effect of the SPSIP block was beyond our prediction. We assumed that we had encountered such an extended sensory blockage, as the fascial plane between the serratus posterior superior-intercostal muscles allows for transition between fascial compartments in transverse, deep-to-surface, and craniocaudal directions. We believe that this innovative approach, which results in sensory blockage of the neck, shoulder, and axilla and caudally to nearly the seventh and eighth thoracic dermatome, can be used in many indications.

The injection of local anesthetic into the interfascial plane between the serratus posterior superior muscle and the rhomboid minor muscle was first described by Takete et al. [10] for the treatment of interscapular myofascial pain. They reported using a volume of 15 mL, which is relatively lower than the volume of LA used in our study [10].

The rhomboid minor muscle attaches to the scapula from the lateral side, and the continuity of the interfascial plane on the superficial serratus posterior to the subscapular region interrupts. These factors would have limited the success of Taketa's technique. However, applying more volume to the fascial planes with transverse and craniocaudal continuity, as we applied between the serratus posterior superior (SPS) muscle and intercostal muscles, provides a more extensive spread of LA and provides sensory blockade in larger areas.

Both the demonstration of dye distribution in cadaveric examination and the dermatome analyses in the patients are significantly different and extensive when compared to other interfascial plane blocks applied to anatomically similar locations. The fact that SPSIP leads to an effective cutaneous block in the neck, shoulder, and hemithorax, even when administered alone, could make this new regional anesthetic approach widely utilized. As with ESPB and RIB, directing the needle towards a bone barrier will reduce the rate of needling-related complications such as pneumothorax. However, this region's anatomy should be fully understood to prepare for any potential complications.

The area for the SPSIP block contains many anatomical structures that need to be identified for safety. The spinal accessory nerve (SAN) travels between the trapezius and rhomboid minor muscles, and local anesthetic can reach this plane [11]. The SAN is a pure motor nerve when blocked, leading to a trapezius motor block with a winged scapula. This condition will be temporary unless direct nerve damage occurs [12]. The motor blockade in the trapezius muscle may contribute to perioperative analgesia in some surgical procedures, such as shoulder and scapula surgery. Although SAN is a pure motor nerve, SAN entrapment is one of the etiological factors in severe shoulder pain, and SPSIP block should be considered as a treatment option.

Similarly, the dorsal scapular artery (DSA) and the dorsal scapular nerve (DSN) course between the rhomboid minor and SPS muscle. DSN is the motor innervation of the rhomboid muscles, and its blockage may also lead to a winged scapula. Although we did not observe any complications in our patients, it is still theoretically possible.

Our study has some limitations. Firstly, we were unable to determine the cephalic extension of the dye, as we did not dissect above the C7 vertebra. Secondarly, we were unable to thoroughly dissect the scapula in the cadaver to determine the subscapular extension of the dye and whether the lateral cutaneous branches or the intercostobrachial nerve was affected. In addition, we did not dissect the supraclavicular fossa and were, therefore, not able to assess the cervical and brachial plexus. Lastly, we did not use SPSIP block for acute postoperative pain or another indication of pain syndromes.

## Conclusions

Our limited preliminary data has shown that SPSIP, a newly defined interfascial plane block, can be used in acute and chronic pain relief. Further randomized controlled trials are needed to verify the exact role SPSIP may play in regional anesthesia and its comparison with different analgesic modalities in surgeries concerning the breast, thorax, and shoulder.
